# The Basics of Artificial Antigen Presenting Cells in T Cell-Based Cancer Immunotherapies

**Published:** 2017-06-26

**Authors:** Lillian R. Neal, Stefanie R. Bailey, Megan M. Wyatt, Jacob S. Bowers, Kinga Majchrzak, Michelle H. Nelson, Carl Haupt, Chrystal M. Paulos, Juan C. Varela

**Affiliations:** 1 Department of Microbiology & Immunology, Medical University of South Carolina, Charleston, SC 29425; 2 Department of Dermatological Surgery and Dermatology, Medical University of South Carolina, Charleston, SC 29425; 3 Department of Surgery, Medical University of South Carolina, Charleston, SC 29425; 4 Department of Hematology and Oncology, Medical University of South Carolina, Charleston, 29425

**Keywords:** Adoptive cell transfer, Artificial antigen presenting cells (aAPCs), T cells, Cancer immunology, Immunotherapy

## Abstract

Adoptive T cell transfer (ACT) can mediate objective responses in patients with advanced malignancies. There have been major advances in this field, including the optimization of the *ex vivo* generation of tumor-reactive lymphocytes to ample numbers for effective ACT therapy via the use of natural and artificial antigen presenting cells (APCs). Herein we review the basic properties of APCs and how they have been manufactured through the years to augment vaccine and T cell-based cancer therapies. We then discuss how these novel APCs impact the function and memory properties of T cells. Finally, we propose new ways to synthesize aAPCs to augment the therapeutic effectiveness of antitumor T cells for ACT therapy.

## INTRODUCTION

The isolation, expansion and infusion of tumor-reactive T cells into patients, called adoptive T cell transfer (ACT), can mediate objective responses in individuals with late-stage tumors [1–3]. Since its development, there have been several advances in this field, including 1) the optimal way to precondition a patient with chemotherapy prior to infusing T cells and 2) how to optimally generate sufficient numbers of T cells using unique cytokines, small molecules and antigen presenting cells (APCs) to instill durable memory responses to tumors. Herein, we focus on the impact of natural and artificial aAPCs in shaping the biology of tumor reactive T cells. We then suggest creative ways to synthesize aAPCs to enhance the persistence, cellular bio-energetic, and antitumor capacity of transferred T cells in patients.

### Adoptive T Cell Transfer

**Figure 1:**
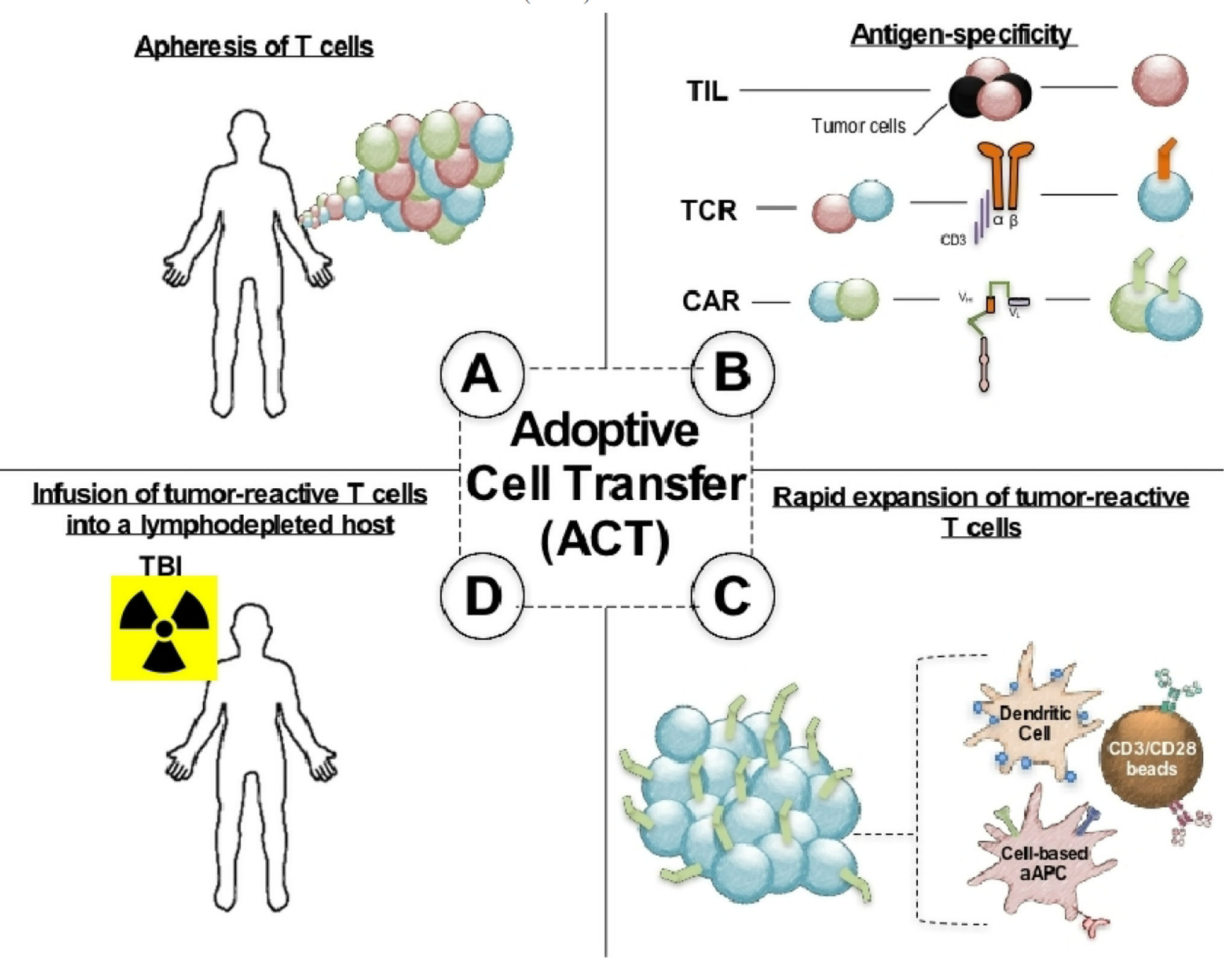
Adoptive T cell therapy approach **A**: Patients’ T cells are isolated from their peripheral blood mononuclear cells (PBMCs) for expansion (both cytotoxic CD8^+^ T cells and helper CD4^+^ T cells). **B**: Isolated T cells are then rendered for antigen-specificity, taken from naturally arising TIL or genetically engineering with a CAR or TCR that recognizes cancer. **C**: Tumor-reactive T cells are then rapidly expanded using dendritic cells or artificial APCs to sufficient numbers. **D**: Expanded T cells are infused to a pre-conditioned host.

Adoptive T cell transfer (ACT) is a customized immunotherapy for patients with advanced malignancies [1,4–6]. This approach involves rapid *ex vivo* expansion of autologous or allogeneic T cells to significant numbers (10e^9–11^), followed by infusion into a pre-conditioned individual, as shown in Figure 1. Also, detailed in Figure 1 are the different types of APCs that may be used to expand T cells *ex vivo*. These APCs include natural dendritic cells (DCs) as well as artificial cell or bead based DCs. T cell products can originate from the tumor (called tumor infiltrating lymphocytes, TILs) or from the peripheral blood. Peripheral blood lymphocytes are rendered antigen-specific by engineering expressions of T cell receptors (TCRs) or chimeric antigen receptors (CARs). Autologous ACT is a promising treatment for individuals with metastatic melanoma with complete response rates in 50% of TIL therapies [3,7–9]. Allogeneic therapies, specifically CD19-CAR-specific transfers, can render objective responses in 83% of patients with acute lymphoblast leukemia (ALL) [5,10,11] and 27% of patients with chronic lymphoblastic leukemia (CLL) [12–14]. A major advancement in adoptive immunotherapy includes host preconditioning prior to cell transfer. The mechanisms underlying the effects of lymphodepletion prior to ACT are discussed below.

### Lymphodepletion Enhances ACT Therapy

ACT clinical trials in the 1990s infused tumor-specific TILs that yielded disappointing responses in melanoma patients [15], mediating objective responses in approximately 30% of patients. However, more than half of patients with advanced melanoma achieved an objective response if they were first preconditioned with a cyclophosphamide/fludarabine lymphodepletion regimen prior to adoptive transfer of TILs [16]. Importantly, some of these patients experienced long-term curative responses with this approach. Finding that host preconditioning augments the antitumor activity of transferred T cells has advanced the field, thus promoting other investigators around the world to perform this therapy in their patients [3,17]. Several mechanisms underlie how lymphodepletion augments ACT therapy, including the elimination of host immune cells that suppress infused TIL. These host cells include host regulatory T cells (Tregs) [18,19] or other host lymphocytes that compete for homeostatic cytokines, such as interleukins 7 and 15 (IL-7 and IL-15) [18,20]. Lymphodepletion also activates the innate immune system through gut microbes that translocate from the injured bowel thereby augmenting the function and persistence of infused T cells [21]. Finally, lymphodepletion ablates MDSCs and regulatory B cells (Bregs) in the tumor microenvironment, which can impair the antitumor activity of infused T cells. Thus, host preconditioning provides an environment where the transferred lymphocytes can engraft and persist in the patient.

Lymphodepletion is not the only factor influencing clinical responses in patients treated with ACT therapy. Emerging findings now show that the ability to expand T cells to sufficient numbers without compromising their antitumor efficacy is a crucial component for successful ACT trials. The importance of how cellular product is expanded and the ideal properties of a therapeutic T cell are key concepts in adoptive immunotherapies. For example, the differentiation status and cellular energetics of tumor-reactive lymphocytes are important for sustaining their durability in the host [22–24]. Below we review recent reports that describe some of the ideal properties of T cells that mediate the highest antitumor responses *in vivo*.

### Central Memory T Cells in Antitumor Immunity

**Figure 2:**
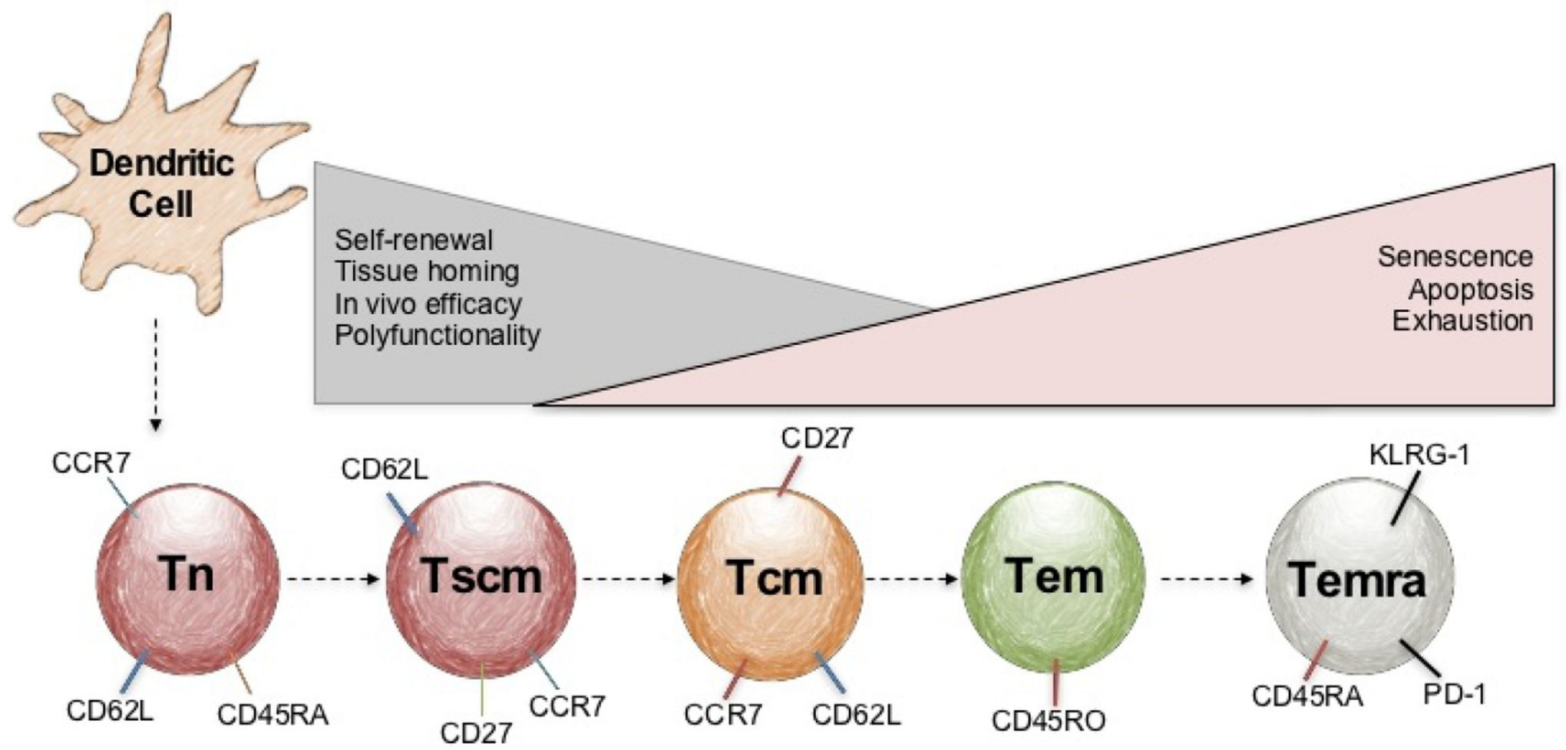
Memory profile of T cells post progressive expansion with DCs T cells progressively differentiate from naïve (CCR7+ CD62L+ CD45RA+), stem (Tscm, CD62L+CD27+CCR7+), central (Tcm, CCR7+CD62L+CD45RO+) to effector (Tem, CCR7-CD62L-CD27+CD45RA-CD45RO+) memory profiles that is influenced by the amount of antigen-stimulation from the dendritic cell or artificial APC and cytokines used for *in vitro* expansion. As a T cell differentiates, they down-regulate certain receptors that can alter their ability to self-renew, home to tissues, secrete cytokines or mount immunity to self or tumor tissue. When a T cell becomes chronically stimulated by antigen they may become terminally differentiated also known as Temra. CD8+ Temra are renderedanergic and/or exhausted denoted by the up-regulation of exhaustion markers PD1 and KLRG1 and re-expression of CD45RA. ACT clinical trials are interested in using stem (Tscm) or central (Tcm) memory T cells for cancer treatment, given their promise in preclinical models compared to the less effective effector memory T cells (Tem, CCR7-CD62L-CD27+CD45RA-CD45RO+).

Lymphocytes naturally progress through differentiation states, which are governed by antigen stimulation from dendritic cells (DCs). It is becoming clearer that T cell’s antitumor efficacy is denoted by the T cell’s differentiation state [25–28]. Their naïve, stem, central and effector memory profile has long been associated with their differentiation state, which can be characterized by the expression of certain surface receptors [25,29,30], as shown in Figure 2. Historically, T cells selected for transfer possessed an effector memory phenotype (CD62L-CD45RA+ expression), with the ability to secrete IFNγ *in vitro* and have *in vivo* cytolytic capacity [26]. Against dogma, Restifo, Gattinoni and co-workers reported that less differentiated stem and central memory CD8^+^ T cells, denoted by their expression of CD62L, CCR7 and β-catenin, were superior at regressing tumors than effector memory CD8^+^ T cells in mice [16,26]. This discovery resulted in part from the finding that tumor-specific CD8+ central memory cells can persist longer *in vivo* than their CD8+ effector memory counterparts [16,22,31]. To further investigate the robustness of central memory T cells, the Dirk Busch lab conducted multiple serial transfer experiments where a mere 100 central memory T cells and 100 effector memory T cells were infused into mice with an infectious disease. They found that the central memory T cells cleared listeria far better than the effector memory T cells [31]. Moreover, in a second and third serial transfer experiment, 100 central memory T cells, but not the 100 effector memory T cells, continued to protect the animal from are-challenge of listeria. Given the ability of ACT with less differentiated T cells to deliver robust antitumor responses in mice, clinical trials are underway to use enriched CD62L^+^ T cells to treat patients with advanced malignancies [32]. Designing an expansion protocol with natural or artificial antigen presenting cells that specifically support the expansion of central over effector memory CD8^+^ T cells might have profound implications for next generation ACT clinical trials. For example, several investigators are exploring the role of TCR “signal strength” improving or hindering the antitumor efficacy of T cells with CD3/CD28 activator beads [33,34], with cell culture plates adhered with anti-CD3 and soluble anti-CD28 [35], or mAbs of CD3 and CD28 [36]. It is becoming clearer that the length of time T cells are initially activated with TCR stimulation, the progression of differentiation occurs, which can negatively prime T cells *in vitro*, decreasing cytokine production and hindering their ability to regress tumor *in vivo* [33–35]. Another key concept about ex vivo T cell activation, are the co-stimulation of CD28 enhancing progressive differentiation through up-regulating glycolysis via the mTOR pathway [36]. The advantages of using aAPCs to prime T cells include two things: 1. Using various costimulatory molecules, other than CD28; like ICOS, to preferentially expand subsets of T cells that will develop a higher antitumor efficacy [33] and 2. Manipulating the duration of aAPCs to activate T cells *in vitro* by length of duration in culture or the amount of beads placed in culture [33,34].

### APC Platforms for the *ex vivo* Expansion of T cells

The development of affordable platforms to expand sufficient numbers of T cells with potent antitumor activity has been a key goal in the field. Initial *ex vivo* T cell expansion protocols used autologous dendritic cells (DCs) that, when co-cultured with T cells, preferentially expanded TILs to treat patients with melanoma [37]. However, the ability to generate enough of antigen-specific T cells with this approach varied between patients, likely due to the fitness of the patient’s T cells and/or DCs [38–41]. There are many reasons why autologous DCs can be challenging to work with. For example, DC-based T cell expansions are complex, requiring multiple cultures, numerous cytokines and extended times for cell expansion. Also, DCs can possess a suppressive phenotype, which does not permit the generation of T cells with a desired phenotype [39–41]. Ultimately these hurdles contribute to complex protocols that are technically complex and costly to reproduce, thus restricting TIL therapies to only a few institutes around the world. These limitations prompted the quest for the generation of clinical grade artificial antigen presenting cells (aAPCs) that could rapidly and simply expand tumor-reactive T cells.

**Figure 3:**
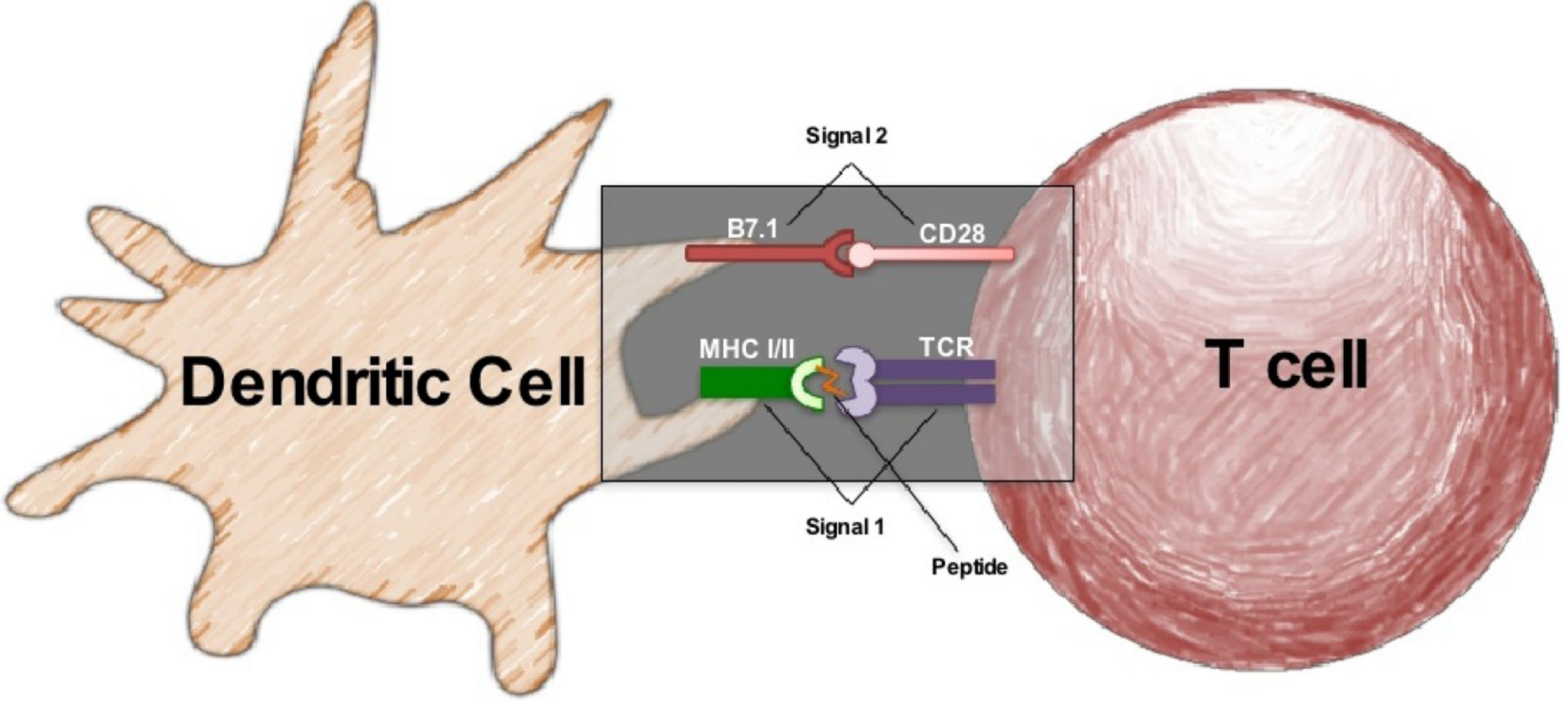
The basics of how a mature dendritic cell activates a T cell via signal 1 and signal 2 Dendritic cells effectively deliver signal 1 by presenting peptide via the MHC to activate the T cell receptor. Signal 2 is critical for complete T cell activation and is done so through ligands specific for the target T cell.

**Figure 4:**
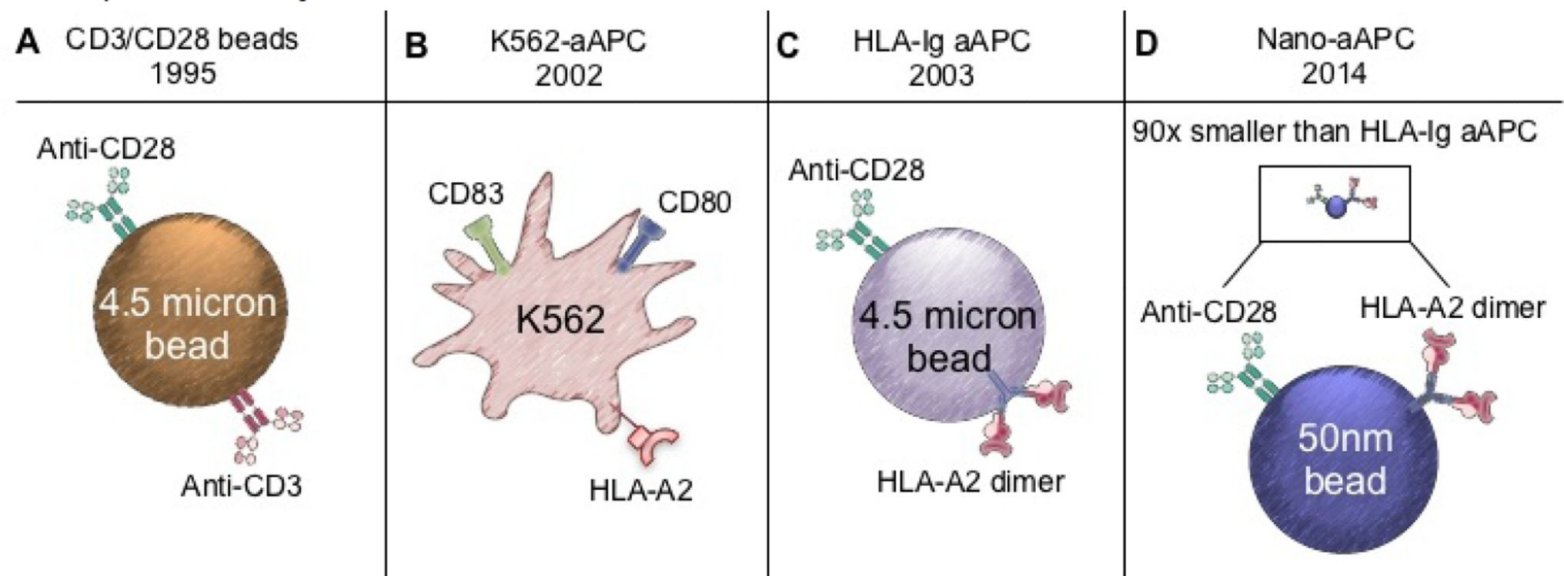
The history of artificial antigen-presenting cells in ACT A)The first artificial APC promoted generic stimulation from a cell-size (4.5 micron) bead coupled with anti-CD3 and anti-CD28 antibodies that rapidly expanded CD4^+^ T cells. B) To support long-term expansion of CD8^+^ T cells, a malignant human cell line was transduced to deliver anti-CD3 and costimulatory antibodies, anti-CD28 or anti-41BB, for preclinical studies. C) To exploit autologous antigen-specific expansion, cell-size (4.5 micron) beads coupled with an HLA-dimer and anti-CD28. D) Further development of the cell-size HLA-aAPC transitioned to nanoscale beads (50 nm) coupled with HLA-A2-dimer and anti-CD28 antibodies.

In the following sections, we discuss how natural DCs (Figure 3) augment TIL based immunotherapy for cancer. We then focus on the evolution of aAPCs (Figure 4) through the years. We discuss immortalized K562 and paramagnetic aAPCs and their role in tumor immunity. The potential of aAPCs is limitless: they can be decorated with any number of co-stimulatory molecules to augment antitumor T cells for ACT therapy.

### Natural Versus Artificial APCs

Dr. Ralph Steinman and his team discovered an APC called a DC in the 1970s and was awarded a Nobel Prize in 2011 for this work [42]. DCs are composed of two distinct lineages: the myeloid and plasmacytoid lineage [43–45]. Immature DCs mature via distinct stimuli in a stepwise fashion. Immature DCs maintain tolerance to self-antigens and blunt immunity to cancer via their expression of various regulatory molecules (such as CTLA-4 or PD-1) and cytokines (i.e. IL-10 and TGF-β). In contrast, mature DCs, activated in response to microbial signals (toll-like receptor ligands), trigger strong effector T cell responses against antigens [44,46]. It is known that DCs are phagocytic cells of the immune system that degrade pathogens and can clear tumors by a process called macropinocytosis [47]. The main role of mature DCs are to sense antigens and produce mediators that activate other immune cells, particularly T cells [48]. DCs are potent stimulators for lymphocyte activation as they express MHC molecules that trigger TCRs (signal 1) and co-stimulatory molecules (signal 2) on T cells [46]. This classic signal 1 signal 2 model: shown in Figure 3, illustrates how a mature DC can activate T cells via TCR/MHC and B7.1/CD28 ligations [44,46]. Additionally, DCs also secrete cytokines that support T cell expansion; many investigators call this signal 3 [49]. Unlike B-cells that can recognize whole antigens, T cells require presented antigen in the form of a processed peptide to recognize foreign pathogens or tumor [46]. Presentation of peptide epitopes derived from pathogen/tumor proteins is achieved through specialized cell-surface glycoproteins called major histocompatibility complex (MHC) molecules. MHC class I (MHC-I) and MHC class II (MHC-II) molecules present processed peptides to CD8^+^ T cells and CD4^+^ T cells, respectively [46]. Importantly, DCs home to inflammatory sites containing abundant T cell populations to foster an immune response [44,50]. Thus, DCs can be a crucial component of any immunotherapeutic approach [51], as they are intimately involved with the activation of the adaptive immune response [45,51].

The ability to generate DCs *ex vivo* from blood monocytes has permitted immunologists to use them clinically as vaccines or in ACT protocols to expand T cells. In the context of vaccines, DC therapy can enhance T cell immune responses to a desired target in healthy volunteers or patients with infectious disease or cancer [37,52]. Treatment with immature DCs, in stark contrast, inhibits CD8^+^ T cell effector responses by propagating regulatory T cells [53]. Thus, DC immunization is of interest to the field of immunotherapy for cancer, infectious diseases and autoimmunity.

### Dentritic Cells in ACT Clinical Trials

Several current protocols for the expansion of tumor-specific T cells use autologous DCs derived from patient’s PBMCs. Immature DCs are activated and matured with the polarizing cytokines GMC-SF and IL-4 [37,52]. Once matured, they are pulsed with a peptide of interest or lysed tumor cells. Mature/antigen-pulsed DCs are then co-cultured with tumor-derived CD8^+^ T cells where they undergo weekly DC re-stimulation until enough TILs are expanded for the desired assay or for therapeutic use [52]. A few clinical trials have successfully treated melanoma patients with expanded TILs using this approach [37,54]. While this therapy can be very effective in treating patients with melanoma, there exist hurdles in this strategy in generating TILs of sufficient quality and quantity. As stated earlier, the limitation of using patient derived-DCs for stimulation and expansion of T cells is that the antitumor responses are not always consistent across donors and that generation of effector memory T cells have diminished function or persistence [39]. For ACT clinical trials, the generation of DCs to reliably expand TILs or CAR T cells are difficult and expensive. The culture process is resource intensive and requires an expensive complex cytokine cocktail. Moreover, there is variability among the donors DCs’ capacity to expand T cells *ex vivo* [40,41]. Perhaps most concerning, DCs are often dysfunctional in patients with cancer [39–41]. Consequently, investigators have spent considerable time and money generating various types of manufactured DCs called aAPCs to better expand T cells with improved responses to antigen. We review some of these aAPCs directly below.

### The K562 Approach: A Cell-based Artificial aAPC

Translational immunologists have successfully expanded human T cells with aAPCs instead of natural APCs. One common approach is the use of the K562 cell line. K562 cells do not express MHC molecules or co-inhibitory/costimulatory molecules, therefore preventing allogeneic T cell responses. However, they do express adhesion molecules (ICAM-1 and LFA-3) needed for effective T cell-APC interactions [55,56]. Additionally, K562 cells secrete MCSF, IL-6, IL-8, TGF-β, and MIP-1α but do not secrete the γ-chain receptor cytokines IFNγ or IL-10 [55]. All advantages aside, this original K562-based aAPC did not meet GMP requirements for clinical use due to unstable transfection of surface molecules that required continuous antibody selection [56]. To address this limitation, several laboratories have improved this aAPC system by genetically redirecting them with a lentiviral vector system to express an array of co-stimulatory molecules and cytokines. The June laboratory generated clinical-grade K562-cell–based aAPCs that could stably express 7 genes using their lentiviral vector system [55,57]. These aAPCs mediated the expansion of human T cells as effectively as natural DCs. These aAPCs were found to be particularly effective at expanding human CD8^+^ T cells. Importantly, the various co-stimulatory ligands engineered on this aAPC could expand terminally differentiated CD28^−^CD8^+^ T cells without the normal requirement of exogenous IL-2 used in nearly all cell culture processes today. This K562-based aAPC has significant promise for designing next generation T cell-based cancer immunotherapies. As shown in Fig. 4B, a clinical grade and GMP-quality K562-based aAPC-A2 line called clone 33 was used to expand MART-1 specific T cells against advanced melanoma [58]. These K562-aAPCs were transfected with four non-retroviral plasmids that encode for HLA-A*02:01 (A2), CD80, CD83, and a puromycin resistance gene (Figure 4B). In comparison to the natural DC expansion platform, aAPC-A2 clone 33 similarly expanded MART-1-specific T cells from both healthy donors and patients with metastatic melanoma (19–49% tetramer positive) [58,59]. Clinical trials are beginning to use this aAPC in combination with various treatment modalities, such as Ipilimumab [58]. However, the K562 aAPC platform has not been widely used for cancer therapy, largely due the fact that these cells are derived from a malignant clone. Although K562-aAPCs are irradiated before co-cultured with T cells so that none of them are detected after T cell expansions, there are appropriate reservations in infusing T cell products with a malignant cell line into cancer patients.

### Dynabeads for Expanding Polyclonal T Cells

To avoid *ex vivo* expansion of human T cells with the K562-aAPC cell lines, ACT protocols have utilized a bead-based aAPC approach for cell expansions. ACT clinical trials expand lymphocytes with paramagnetic beads coated with CD3 and CD28 agonist antibodies (called activator beads). Fig. 4A illustrates the bead construct of simultaneously delivering both signal one (anti-CD3) and signal two (anti-CD28) for non-specific stimulation that mediates robust expansions of human T cells for up to several weeks [60,61]. This approach reproducibly drives multiple rounds of proliferation of T cells, and can result in greater than 1 × 10^9^-fold expansion of the input cell population [62]. This large expansion is due, at least in part, to the CD28-mediated induction of telomerase in CD4+ T cells [62,63]. Despite extensive *ex vivo* replication, bead-expanded T cells retain *in vivo* proliferative capacity. Furthermore, it was discovered that these anti-CD3/28-coated beads also promoted vigorous expansion of CD4^+^ T cells from patients with HIV. Interestingly, during expansion the number of HIV-positive CD4^+^ T cells declined overtime to nearly undetectable levels [60]. This important discovery led to the manufacturing of GMP-compliant anti-CD3/CD28 beads and the first Phase I clinical trial conducted by the June and Riley lab at the University of Pennsylvania [61]. Since then, anti-CD3/CD28-coated beads have been extensively used to expand T cells for use in multiple clinical trials. For example, these beads are used to expand T cells that are genetically redirected to express chimeric antigen receptors that recognize CD19-postiive hematological malignancies (i.e. CD19-CARTs) [5,64,65]. In Phase 1 clinical trials, patients receiving CD19-specific CAR therapies have rendered outstanding objective response rates of 93% in ALL, 63% in CLL, and 36% in lymphoma [5,6,67]. While these CD3/CD28 activator beads deliver robust expansion of engineered tumor-reactive T cells, development of antigen-specific expansion platforms to transfer autologous tumor-specific T cells is a long-term goal within the field. Discussed below are novel bead-based aAPCs that can rapidly expand antigen-specific T cells from healthy donors.

### Harnessing Antigen Specific Activation with aAPCs

Besides TIL stimulation with autologous dendritic cells, earlier attempts to generate antigen-specific T cells with artificial APCs included either cell-based approaches with the *Drosophila spp*. cell line, the K562 cell line or exosomes coated with MHC class I peptides and B7.1/2 (a natural ligand for CD28) molecules [56,57,68]. In 2003, Oelke and colleagues developed a bead-based approach to expand antigen-specific T cells, shown in Fig. 4C. This aAPC is a magnetic bead of cell-size (4.5 micron) coated with HLA-A2-Ig dimer molecules (signal 1) and anti-CD28 antibodies (signal 2) [69–71]. HLA-Ig aAPCs expanded CMV- and MART-1-specific T cells 10^6^-fold in less than two months [69]. Additionally, bioluminescence technology revealed that MART-1 specific T cells expanded with HLA-Ig-based aAPCs trafficked to the HLA-A2+ but not to HLA-A2-melanoma tumors [72]. Furthermore, the tumor growth was inhibited, confirming that these T cells eradicated tumor in an antigen specific manner [72]. This technology progressed to a nanoscale platform, offering new advantages in how immunologists expand antigen-specific T cells for cancer therapies [73,74].

### Nanoscale Expansion Platforms for ACT

Recent evidence suggests that nanosize-aAPCs (50 nm), which are 90-times smaller than traditional CD3/CD28 beads (4.5 um) can be more advantageous at expanding T cells *ex vivo*. First, these beads mimic natural biology, as the initial TCR engagements on T cells occur at nanoscale clusters that could enhance antigen-specific activation [73–76]. The size of the nano-aAPCs may be able to select T cells with a low precursor frequency in the tumor and blood [76,77]. The Oelke lab’s nanoscale aAPC successfully expanded antigen-specific T cells *ex vivo* with high antitumor activity *in vivo* [74]. These nanoscale aAPCs are biocompatible iron-dextran paramagnetic nanoparticles (50 nm) or are avidin-coated quantum dot nanocrystals, (30 nm) [74]. Each type of nano-aAPC is coupled with MHC-Ig (or HLA-Ig) dimers, K^b^-Ig and D^b^-Ig (signal 1) and CD28 antibodies (or other costimulatory agonists) for signal 2: shown in Figure 4D. These nano-aAPCs were shown to expand highly functional SIY-specific or gp100-specific T cells after re-stimulation as well as mediate comparable Pmel (gp100-specific) expansions to the micro-scale aAPCs [74]. Additionally, nano-aAPCs inhibited B16 melanoma tumor growth in mice by expanding antigen-specific T cells with function and persistence *in vivo* [74]. Importantly, this preclinical finding can be translated to human T cell assays, as nano-aAPCs also mediated an 800-fold expansion of human T cells that could recognize and lyse influenza [74].

**Figure 5:**
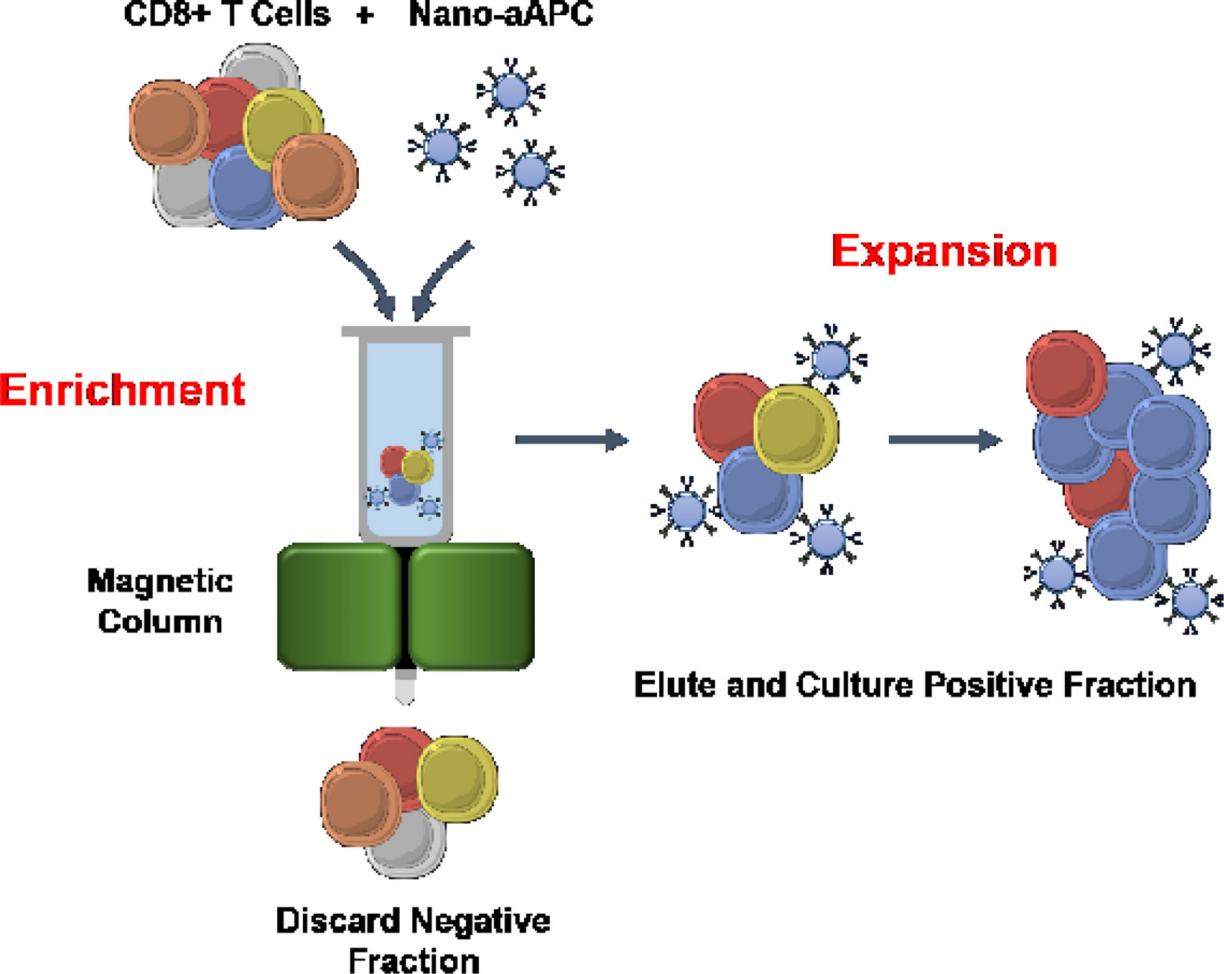
Enrichment and Expansion approach (E+E) for antigen-specific T cells **Enrichment (“Adapted with permission from K. Perica *et al.*, Enrichment and Expansion with Nanoscale Artificial Antigen Presenting Cells for Adoptive Immunotherapy. *ACS Nano* 9, 6861–6871 (2015). Copyright (2015) American Chemical Society.”):** CD8+ T cells from HLA-A2+ donors are incubated with nano-beads (50 nm) coupled with HLA-Ig dimer and CD28 antibodies. After incubation, the beads and cells are washed through a magnetic column. **Expansion:** the positively selected cells are eluted from the column and cultured for 14 days. The E+E method enriched and expanded naïve MART-1, NYESO-1, and CMV-specific T cells from healthy donors to a central memory phenotype and were multifunctional.

To expand rare antigen-specific precursors that lyse tumors, such as NY-ESO-1 and WT-1-reactive T cells, novel enrichment and expansion (E+E) protocols have been reported [73]. Figure 5 demonstrates the E+E method, where first, antigen-specific CD8+ T cells from HLA-A2+ donors are incubated with paramagnetic nanoparticles decorated with HLA-Ig-dimers pulsed with MART-1 peptide (for example) and anti-CD28. This culture is then enriched for antigen-specificity through a magnetic column, where positively selected cells are cultured for 14 days [73]. This approach mediates robust expansions for MART-1 and NYESO-1-specific T cells [73]. Additionally, this novel aAPC platform can expand neoantigen-specific T cells using predicted neo-epitopes obtained from a sequenced tumor [73]. This E+E platform could make a substantial contribution to next generation ACT trials, where rare yet very effective T cells can be expanded with a durable memory phenotype before being re-infused into a properly preconditioned patient with cancer.

### Closing Remarks and Future Directions

Compared to naturals DCs, aAPCs are proven to be a simpler and more cost-effective method for expanding genetically engineered and antigen-specific T cells for adoptive cellular therapy. aAPC platforms allow endless combinations of signal 1 and 2 for expanding the optimal T cell for specific malignancies. The evolution of aAPC platforms bring clinicians one-step closer to harnessing the power and ability of our own immune system to fight off even the most detrimental diseases. Currently, researchers are discovering novel ways to obtain robust T cell expansions of high quality and quantity by using various inhibitory drugs and manipulations used in cell cultures. Preclinical studies using the PI3Kδ inhibitory drug, CAL-101, for individuals with CLL, are being explored as a treatment modality [78], as well as, supplementation for T cell cultures. Another alternative involves the use of various costimulatory molecules on aAPCs. Researchers are exchanging CD28 for the costimulatory molecules ICOS or 41BB to explore potential T-cell potency. Emerging studies are revealing the therapeutic effectiveness of Th17 cells in preclinical mouse models. A subset of CD4+ T cells once thought to be a controversial lineage for cancer immunotherapies is now a potentially advantageous subset for adoptive transfer due to their cytolytic capacity, ability to have self-renewal properties, and ability to persist [79]. When Th17, and even IL-17-producing CD8+ T cells (Tc17), are expanded with ICOS, their antitumor efficacy increases compared to co-stimulation with CD28 [80,81]. Other emerging concepts in the world of aAPCs are the methods to enrich autologous antigen-specific T-cells from cancer patients as a potential cell transfer therapy. As described earlier, the nano-scale aAPC platform is a novel approach to enrich antigen-specific T-cells with an HLA-A2+ antigen-presentation [73]. Further preclinical studies in our lab are investigating the optimal signal 1, comparing dimers versus tetramers to enrich antigen-specific T cells. Whether this approach can effectively expand tumor-specific T cells with a less differentiated phenotype and maintain functional capacity is yet to be known. The developments of aAPCs have been improved significantly since the 1995 CD3/CD28 beads. Each aAPC provides advantages over the other, as well as, limitations, as shown in Table 1. Further investigations are underway to achieve optimal aAPC protocols to generate durable memory T cells for broad use in the clinics.

## Figures and Tables

**Table 1:** Advantages and limitations of current aAPCs.

Approach	aAPC Foundation	Advantages	Limitations	Comments

**Cell-based**				

**K562**	Human erythroleukemia	Antigen-specific & polyclonal T cell expansions Supports long-term CD8+ expansions	Malignant cell line Cannot be removed from cultures	Not used in ACT clinical trials

**Bead-based**				

**CD3/CD28 activator beads**	Paramagnetic, polystyrene	Supports long-term CD4+ expansions Rapid expansions to 10^9^ Can substitute CD28 for 41BB/IC	Only polyclonal expansions Removal from culture necessary	Used in CAR-T clinical trials

**HLA-Ig beads**	Paramagnetic, polystyrene	Rapid antigen-specific - expansions Can use other HLA-Ig molecules	Removal from culture necessary	Optimized to nano-sized beads for rare precursor T cell expansions

**Nano-aAPC**	Paramagnetic, iron dextran, nanoparticles	Antigen-specific and polyclonal expansions Allows infusion into patients	Quality control methods are expensive	Optimizing in progress Neo-antigen expansions
